# Niobium pentoxide-mediated engineering of the SnO_2_/perovskite interface: simulation study

**DOI:** 10.1039/d6ra10070h

**Published:** 2026-11-04

**Authors:** Mustafa Kareem, Basem Abu Zneid, Anjan Kumar, Gopalakrishna K., Satish Choudhury, Ramneek Sidhu

**Affiliations:** a College of Remote Sensing and Geophysics, Al-Karkh University of Science Haifa St. Baghdad 10011 Iraq; b College of Science, University of Warith Al-Anbiyaa 56001 Karbala Iraq dr.mustafa@kus.edu.iq; c Faculty of Technical Education, Hourani Center for Applied Scientific Research (HCASR), Al-Ahliyya Amman University Amman Jordan; d Department of Electronics and Communication Engineering, GLA University Mathura-281406 India; e Department of Electronics and Communication Engineering, School of Engineering and Technology, JAIN (Deemed to be University) Bangalore Karnataka India; f Department of Electrical & Electronics Engineering, Siksha ‘O' Anusandhan (Deemed to be University) Bhubaneswar Odisha-751030 India; g Department of Electronics and Communication Engineering, Chandigarh University Mohali Punjab India

## Abstract

Planar perovskite solar cells suffer from interfacial losses at the perovskite/charge transport layer junction, limiting their efficiency and stability. This work introduces an ultrathin niobium pentoxide layer between the electron transport layer and perovskite interface, acting as an interlayer. The niobium pentoxide interface enables charge transport across the interlayer in the solar cells without causing any significant changes in the characteristics of the perovskite absorber. This modification suppressed leakage current and charge carrier recombination, decreasing the energy barrier for electron extraction, leading to improved open-circuit voltage and overall power conversion efficiency. The method achieved a maximum efficiency of 25.19% in single-junction typical perovskite solar cells in comparison to the 22.66% for the optimized device without modification. Moreover, increasing operating temperature from 300 K to 400 K reduced the normalized efficiency of the solar cell only by 14% of its initial performance at 300 K, indicating a low reduction in electrical device properties. This study offers insights into engineering the interface properties and improving the photovoltaic performance of perovskite photovoltaics.

## Abstract 2

This work introduces an ultrathin niobium pentoxide layer between the electron transport layer and perovskite interface, acting as an interlayer.

## Graphical abstract

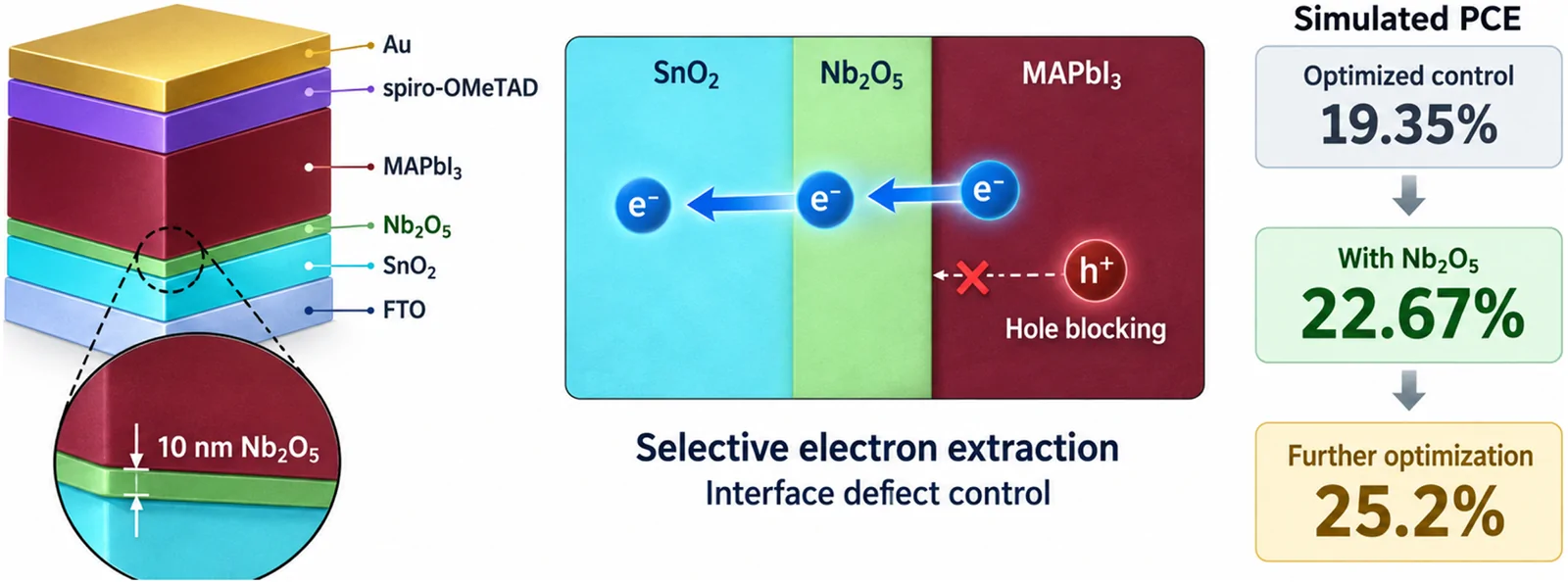

## 1. Introduction

In two decades, the efficiency of metal halide perovskite solar cells (PSCs) has reached a verified 27.98% after extensive research and development.^1^ This advancement can be mainly assigned to the superior photoelectrical characteristics inherent to perovskite semiconductors and the solution processability of PSCs.^2^ Despite such rapid enhancement of their performance, the concern of stability, the critical barrier preventing their commercialization, has yet to be resolved. As photovoltaics are operated in the ambient environment, they must withstand heat, UV radiation, and moisture.^3^ Besides, the instability of PSCs can be caused not only by the intrinsic instability of the perovskite layer but also by interfacial reactions arising at different layers.^4^ First-principles studies have recently broadened the scope of perovskite-derived materials beyond conventional lead-halide PSCs for optoelectronic applications. These studies have explored the structural stability, electronic structure, optical response, and transport properties of hydride perovskites, lead-free halide double perovskites, and oxide-based double perovskites and have shown the influence of chemical composition on their functional properties.^5–8^

The interface quality between perovskites and the tin oxide (SnO_2_) electron-transporting layer is known to suppress the device performance because of increased leakage current and nonradiative recombination.^9^ Interfacial engineering was used to regulate the trap-state density of the perovskite, enhance charge carrier generation/collection, and mitigate residual stress within the perovskites.^10^ Therefore, interface modification is significant for improving both the power conversion efficiency (PCE) and stability of perovskite devices. Niobium pentoxide (Nb_2_O_5_) is a promising n-type semiconductor demonstrating a wide optical bandgap and high electron mobility, and more importantly, enhanced chemical stability at the ETL/perovskite junction.^11^ The conduction band edge of Nb_2_O_5_ is typically considered to be close to that of methylammonium lead iodide (MAPbI_3_) perovskite.^12^ Previous studies have demonstrated Nb_2_O_5_ film as an efficient hole-blocking layer of surface recombination in dye-sensitized and silicon solar cells.^13,14^ Interestingly, Kogo *et al.* and Fernandes *et al.* have reported the incorporation of Nb_2_O_5_ as a compact hole-blocking layer in perovskite devices independently.^15,16^ Zhang *et al.* modified the zinc oxide ETL surface by spin-coating a thin Nb_2_O_5_ as a surface passivation layer in planar PSCs. The modified ETL with Nb_2_O_5_ formed a dense and highly crystalline MAPbI_3_ film, and the corresponding PSC yielded a maximum PCE of 14.57%.^17^ Later, Chavan *et al.* deposited an Nb_2_O_5_ interlayer at the titanium oxide (TiO_2_)/perovskite interface by atomic layer deposition. The ultrathin Nb_2_O_5_ film improved the electronic features and morphology of the fabricated perovskite layers with reduced interfacial recombination losses, thus improving PCE to 21.04%.^12^ Recently, Sharmila *et al.* passivated the TiO_2_/perovskite interface by doping the Nb_2_O_5_ film with silver (Ag) in mesoporous PSCs. This approach improved charge transport pathways by suppressing interface-related recombination losses. The best-performing Ag:Nb_2_O_5_-based PSC attained an efficiency of 14.33%.^18^ However, the benefits of Nb_2_O_5_ as an interlayer at the SnO_2_/MAPbI_3_ interface need further explanation.

Herein, we explore the potential of an ultrathin Nb_2_O_5_ surface passivator between the MAPbI_3_ absorber and SnO_2_ layer to facilitate selective electron transport. The simulated perovskite device with the Nb_2_O_5_ interlayer showed lower recombination losses and higher electric field distribution at the ETL/perovskite interface compared to the unmodified device. The simulated band structure diagram revealed a preferable energy-level alignment for the Nb_2_O_5_- engineered PSC due to the reduction of conduction band offset (CBO) at the ETL/perovskite junction. Consequently, the optimized Nb_2_O_5_-based PSC achieved a PCE of 25.19% with an enhanced open circuit voltage (*V*_OC_) of 1.248 V compared to the 22.66% for the device without interlayer. Our results in this research not only exhibit the potential of the Nb_2_O_5_ interlayer for high-performance PSCs but also provide a systematic strategy for device optimization.

## 2. Methodology

Solar cell architecture was proposed utilizing the Solar Cell Capacitance Simulator (SCAPS-1D, version 3.3.07) simulation program, established by Burgelman *et al.* at the University of Ghent.^19^ SCAPS-1D software solves two fundamental semiconductor equations, including the Poisson equation and the continuity equation of electrons and holes under steady-state conditions. Poisson's equation governs the electrostatic potential (*ɸ*) inside a solar cell:^19^1
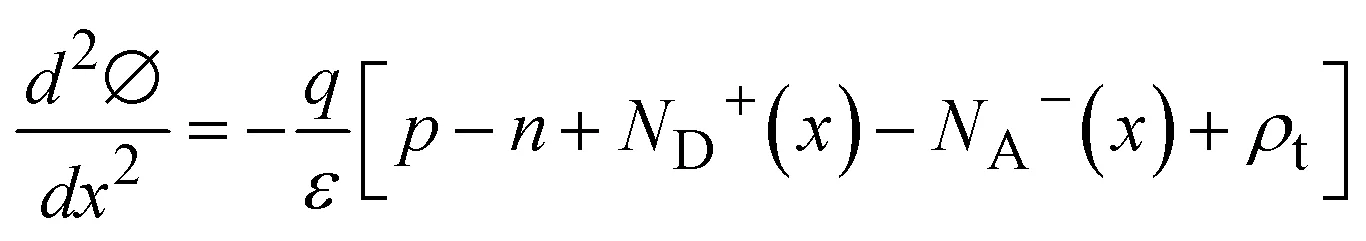
where *ε* is the dielectric permittivity, *q* is the elementary charge, *n* and *p* refer to the electron and hole densities, *N*_D_^+^ and *N*_A_^−^ denote donor and acceptor concentrations, respectively, and *ρ*_t_ corresponds to the trapped charge density.

Continuity equations describe carrier transport as follows:2
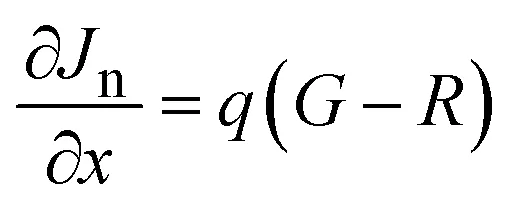
3
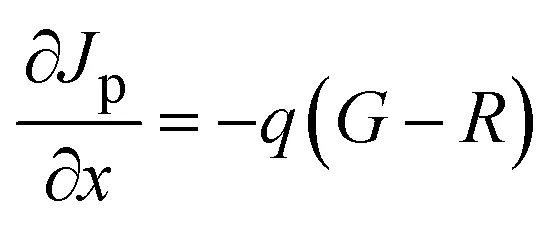
where *J*_n_ and *J*_p_ represent the electron and hole current densities, while *G* and *R* represent the generation and recombination rates, respectively.

The drift-diffusion model expresses the carrier current densities:4
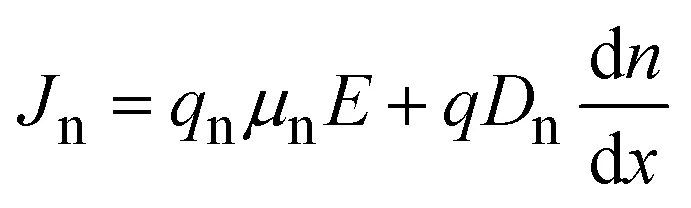
5
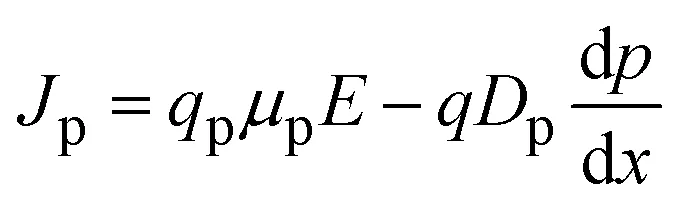
where *µ*_n_ and *µ*_p_ are the electron and hole mobilities. *D*_n_ and *D*_p_ are the diffusion coefficients 
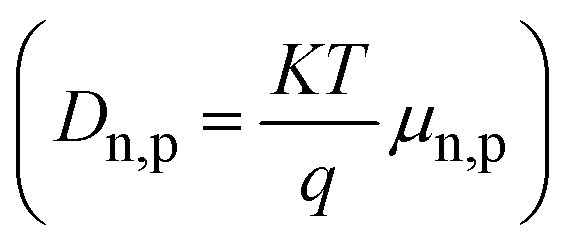
 and *E* is the electric field.

The absorption coefficients of the input layers were calculated by the SCAPS-1D optical absorption model with the square root model 
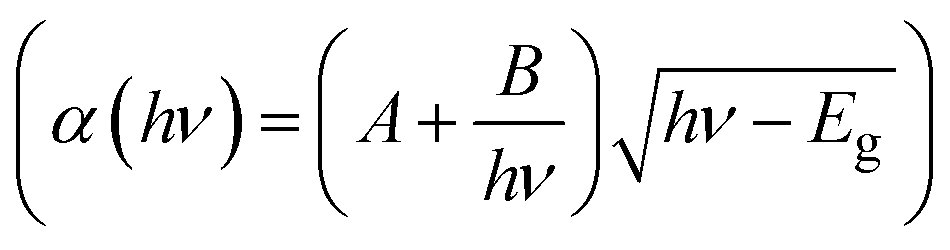
 where *α* is the absorption coefficient (cm^−1^), *A* and *B* are material-dependent empirical constants, *h* is Planck's constant, h*v* is the incident photon energy (eV), and *E*_g_ is the optical bandgap (eV).^20^ All simulations were carried out under illumination of 1000 W m^−2^ at AM 1.5 G, an operating temperature of 300 K, a series resistance (*R*_S_) of 2.0 Ω cm^2^, and a shunt resistance (*R*_SH_) of 10^3^ Ω cm^−2^. We used fluorine-doped tin oxide (FTO) as an illuminated side with a work function of −4.4 eV, whereas gold (Au) was applied as a rear electrode with −5.1 eV work function. In order to propose a reliable solar cell structure, the defect densities in bulk and at the interfaces (the ETL/perovskite and perovskite/HTL boundaries) were added (see Tables 1 and 2). Gaussian energy distribution with a characteristic energy of 0.1 eV was considered for all defect states. The thermal velocities of the electrons and holes were maintained at 10^7^ cm s^−1^ during simulation. This simulation tool was employed to analyze the different performance parameters, including current density–voltage (*J*–*V*) characteristics, quantum efficiency (QE), generation/recombination rate, band structure diagram, and impedance spectrum of a PSC.

**Table 1:** The key parameters of the materials utilized in the PSC simulation

Parameters (units)	SnO_2_	Nb_2_O_5_	MAPbI_3_	Spiro-OMeTAD
Thickness [nm]	75	10	370	100
Bandgap [eV]	4.1	3.4	1.59	3.02
Electron affinity [eV]	4.39	4.1	3.9	2.18
Dielectric permittivity	7.25	41	25	3.0
Density of states in conduction band [cm^−3^]	2.2 × 10^18^	8.93 × 10^19^	1.0 × 10^19^	2.2 × 10^18^
Density of states in valence band [cm^−3^]	1.8 × 10^19^	1.0 × 10^19^	1.0 × 10^18^	2.0 × 10^16^
Electron mobility [cm^2^ V^−1^ s^−1^]	265	20.73	0.66	7.9 × 10^−3^
Hole mobility [cm^2^ V^−1^ s^−1^]	7.6	1.0 × 10^−3^	0.43	7.9 × 10^−3^
Shallow donor density [cm^−3^]	2.4 × 10^19^	7.5 × 10^18^	—	—
Shallow acceptor density [cm^−3^]	—	—	1.0 × 10^16^	1.2 × 10^17^
Total defect density [cm^−3^]	1.0 × 10^16^	1.0 × 10^16^	1.03 × 10^16^	1.0 × 10^15^
Electron capture cross-section [cm^2^]	1.0 × 10^−15^	1.0 × 10^−15^	1.8 × 10^−16^	1.0 × 10^−15^
Hole capture cross-section [cm^2^]	1.0 × 10^−15^	1.0 × 10^−15^	1.8 × 10^−16^	1.0 × 10^−15^
References	21–24	14, 25 and 26	27	28–30

**Table 2:** The interfacial parameters of the simulated PSCs

Parameters/interfaces	SnO_2_/Nb_2_O_5_	SnO_2_/MAPbI_3_	Nb_2_O_5_/MAPbI_3_	MAPbI_3_/spiro-OMeTAD
Defect type	Neutral	Neutral	Neutral	Neutral
Capture cross section for electrons [cm^2^]	1.0 × 10^−19^	1.0 × 10^−19^	1.0 × 10^−19^	1.0 × 10^−19^
Capture cross section for holes [cm^2^]	1.0 × 10^−19^	1.0 × 10^−19^	1.0 × 10^−19^	1.0 × 10^−19^
Energetic distribution	Gaussian	Gaussian	Gaussian	Gaussian
Reference for defect energy level *E*_t_	Above the highest *E*_V_	Above the highest *E*_V_	Above the highest *E*_V_	Above the highest *E*_V_
Energy with respect to reference [eV]	0.60	0.60	0.60	0.60
Total defect density [cm^−2^]	1.0 × 10^13^	1.0 × 10^13^	1.0 × 10^13^	1.0 × 10^13^

## 3. Results and discussion

Fig. 1a exhibits the reference FTO/SnO_2_/MAPbI_3_/spiro-OMeTAD/Au device structure proposed by SCAPS-1D software. As shown in Fig. 1b, the simulated *J*–*V* characteristic agrees well with the experimentally reported device.^31^ The proposed device structure used in this simulation is identical to that reported in the literature, except for the replacement of the front electrode (ITO) with FTO. The simulation based on initial data reveals a PCE of 14.04% with a *V*_OC_ of 1.034 V, a short-circuit current density (*J*_SC_) of 20.61 mA cm^−2^, and a fill factor (FF) of 65.82% compared to PCE = 14.58%, *V*_OC_ = 1.08 V, *J*_SC_ = 20.08 mA cm^−2^, and FF = 66.80% for the experimental device. This consistency supports the SCAPS-1D model reliability to calibrate and reproduce the key photovoltaic parameters. Fig. 1c illustrates the band structure diagram of the reference PSC under AM 1.5 G illumination. The diagram reveals a relatively large band bending at the SnO_2_/MAPbI_3_ valence band and the MAPbI_3_/spiro-OMeTAD conduction band. The deep valence band of the SnO_2_ ETL prevents the back transfer of holes to the front electrode, while the high conduction band of the spiro-OMeTAD HTL makes a barrier to electron leakage to the back contact, thus improving carrier selectivity. However, the ETL/perovskite junction exhibits a large CBO edge (0.49 eV), which raises interface recombination and lowers *V*_OC_ due to the notable discrepancy between their electron affinities. Moreover, the simulated device shows pronounced electric fields at the interfaces (Fig. 1d), suggesting efficient photoexcited carrier separation. Notably, the reference device demonstrates high photocurrent because of the strong absorption coefficient of the MAPbI_3_ absorber at the entire visible range (Fig. 1e). The high optical absorbance and effective carrier collection reflect a high simulated QE spectrum, as depicted in Fig. 1f.

**Fig. 1:**
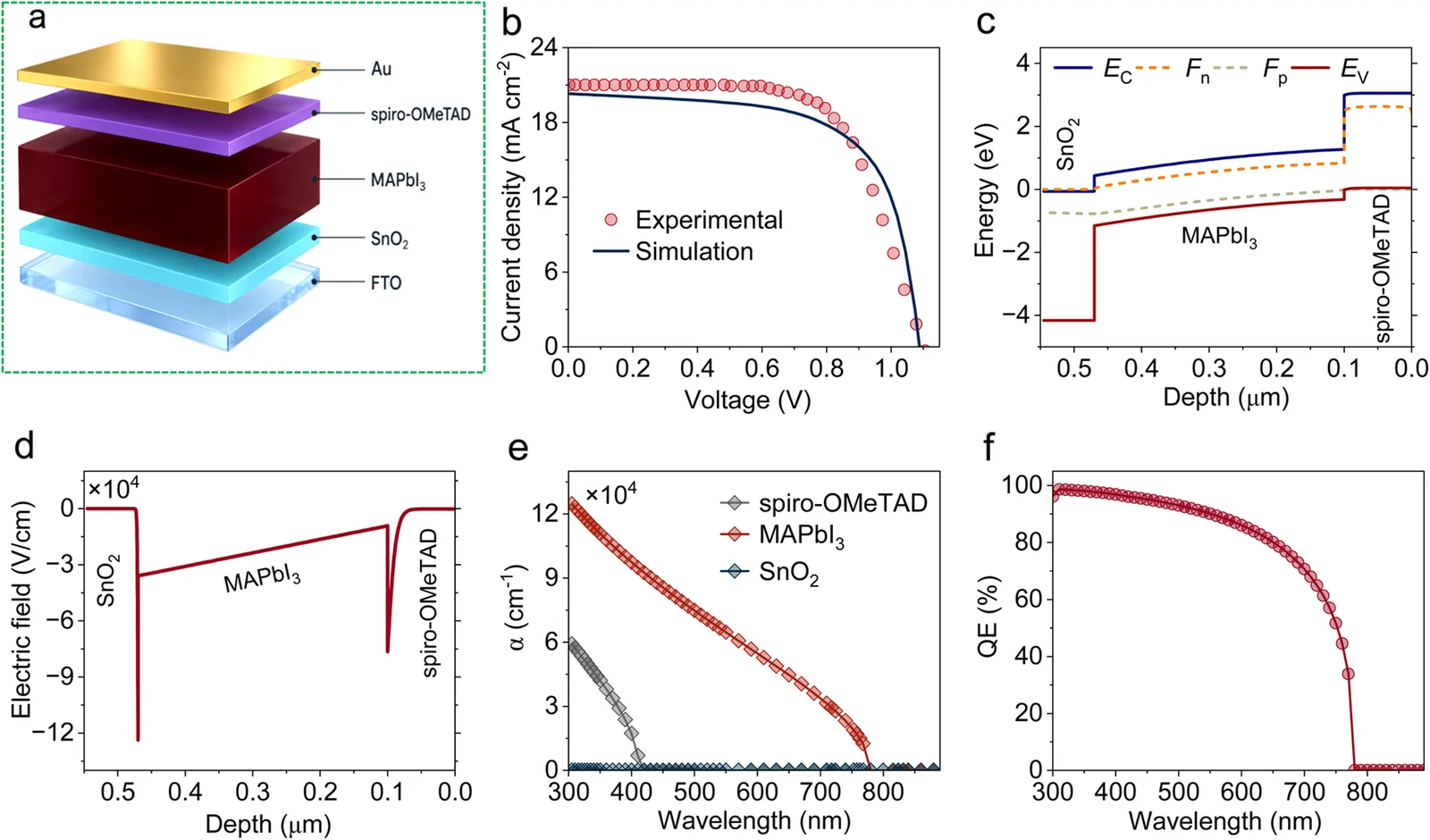
Schematic illustration and calibration of the typical PSC. (a) The layered structure of FTO/SnO_2_/MAPbI_3_/spiro-OMeTAD/Au. (b) Calibration *J*–*V* curve of the proposed PSC experimental device.^31^ (c) Energy level alignment of PSC under AM1.5 light. (d) Electric field distribution. (e) Absorption coefficient curves. (f) QE spectrum.

Fig. 2a–d show the photovoltaic parameters of reference PSCs with varying perovskite layer thickness and bulk defect density. Increasing perovskite thickness primarily enhances *J*_SC_ value from 16.222 mA cm^−2^ to 23.794 mA cm^−2^ owing to improving optical absorbance efficacy at low-defective perovskites. Also, the *V*_OC_ parameter reveals a modest increase from 0.99 V to 1.046 V with 0.8 µm perovskite thickness and 10^14^ cm^−3^ defect density because a thick absorber enhances photogeneration of carriers and low defects reduce trap-assisted recombination 
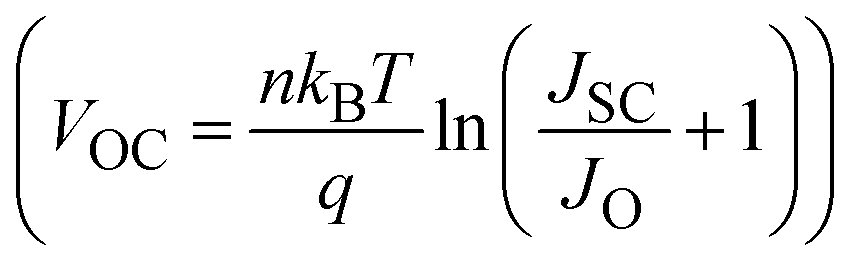
.^32^ This synergized influence improves quasi-Fermi-level splitting (QFLS), thus giving a rise to the *V*_OC_. Nevertheless, highly defective perovskites degrade FF because the increasing absorption and carrier generation are unable to compensate for the recombination losses. Such conditions suppress the diffusion length and lifetime of photoexcited carriers, hence minimizing the number of electrical charges to reach the electrodes. The best-performing device achieves the highest PCE of 18.12%. Fig. 2e exhibits the *J*–*V* plots of PSCs at low and high defect densities while keeping layer thickness constant at 0.8 µm. A high-defect level clearly deteriorates device performance, particularly *J*_SC_ and FF parameters. QE spectra further confirm the photocurrent reduction at high defect density due to damaged collection of photocarriers (Fig. 2f). Fig. 2g shows the impedance spectra of the simulated PSCs under AC bias. The impedance analysis was extracted from capacitance–frequency (*C*–*f*) characteristics under small-signal AC analysis under an illumination intensity of 100 mW cm^−2^ (AM1.5 G, 1 sun), at a temperature of 300 K and a DC bias voltage of 0 V. The frequency was varied from 1 Hz to 10^6^ Hz to investigate the frequency-dependent electrical response of the solar-cell configurations. The device with low defect density reveals an enlarged Nyquist curve, suggesting higher recombination resistance. This trend is further consistent with the recombination rate profiles shown in Fig. 2h. The recombination rate notably reduced within the bulk perovskite layer at low defect levels, supporting decreased carrier losses.

**Fig. 2:**
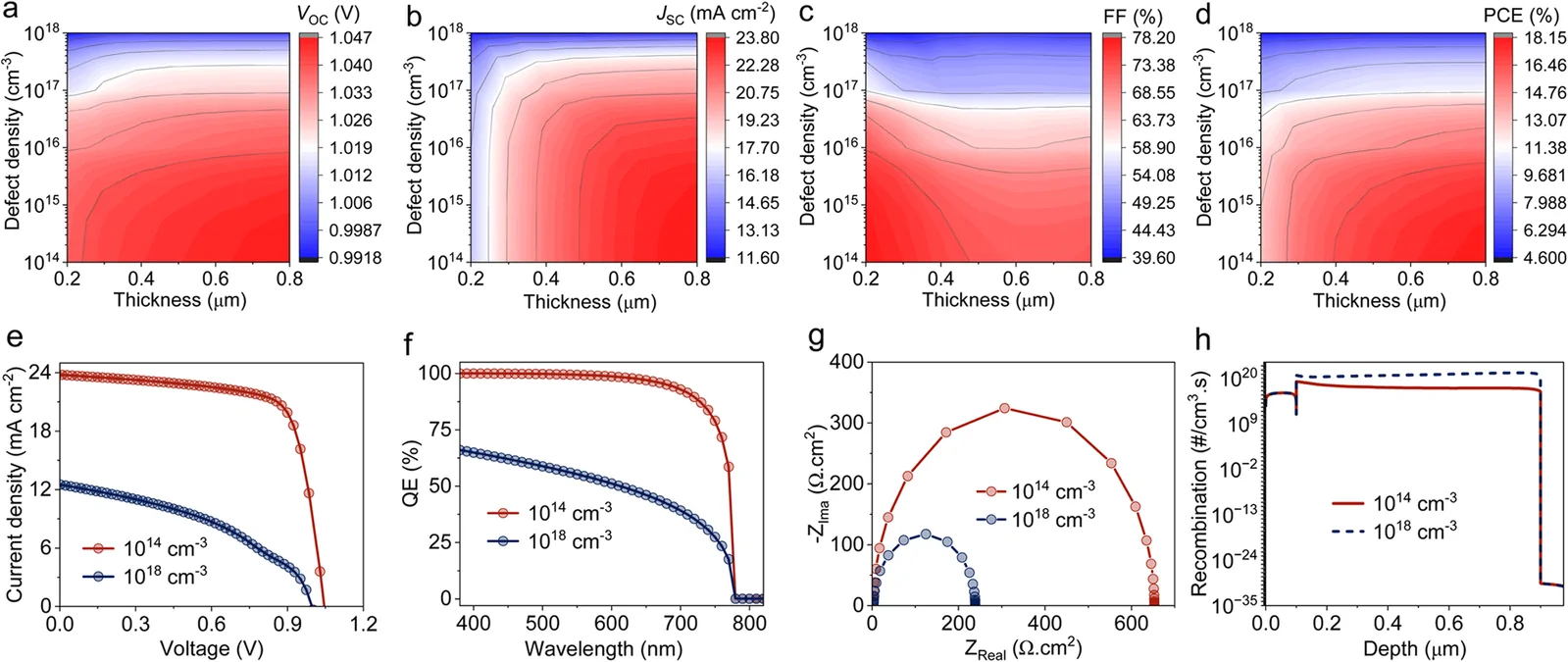
The coupling influence of total defect density and layer thickness of MAPbI_3_ absorber on solar cell performance. The contour plots for (a) *V*_OC,_ (b) *J*_SC_, (c) FF, and (d) PCE of PSCs. (e) The *J*–*V* curves of devices with low and high defect densities at a constant thickness of 800 nm. (f) QE spectra. (g) Nyquist curves. (h) Recombination rate profiles.

The combined influence of shallow acceptor density and hole mobility of perovskite on device parameters is depicted in Fig. 3a–d. At low doping densities, higher hole mobilities result in remarkable improvement in all solar cell parameters. This improvement can be related to an increase in the hole conductivity and reduced charge transfer time, so photoexcited carriers can reach electrodes before recombining. However, increasing acceptor concentration for the devices with low carrier mobility slightly enhances photovoltaic parameters due to increasing the available hole population. Then, increasing hole mobility strongly limits the influence of acceptor density and can slightly reduce PCE. The optimized PSC attains a PCE of 19.31% at a low doping level (10^13^ cm^−3^) and high hole mobility (20 cm^2^ V^−1^ s^−1^).^33^ As shown in Fig. 3e, increasing hole mobility is largely improving *J*_SC_ from 10.1 mA cm^−2^ to 24.1 mA cm^−2^ and FF from 28% to 76.66%, which leads to enhancing *J*–*V* characteristics and device efficiency. The *J*_SC_ reduction is further confirmed by suppressing the QE spectrum (Fig. 3f) due to limiting carrier generation/collection. Additionally, Fig. 3g reveals a significant increase in the semi-circular curve at high hole mobility owing to improving recombination resistance. This pattern is further supported by the recombination rate profile in Fig. 3h. The device with high hole mobility shows mitigated recombination processes in bulk perovskite and ETL, which is related to altered transport dynamics.

**Fig. 3:**
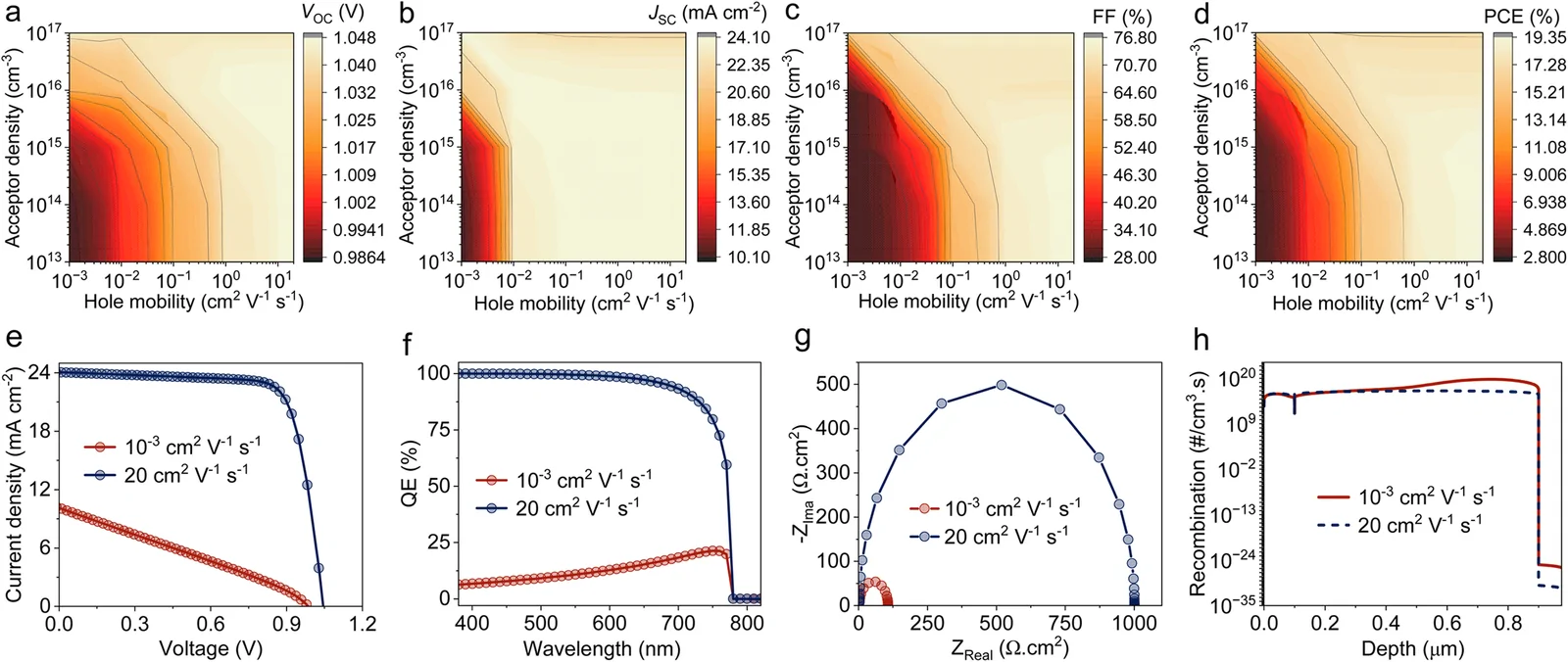
Analysis of the effect of hole mobility and shallow acceptor density of MAPbI_3_ absorber on device performance. The contour maps for (a) *V*_OC_, (b) *J*_SC_, (c) FF, and (d) PCE of devices. (e) The *J*–*V* plots of cells with low and high hole mobilities at a constant acceptor density of 10^13^ cm^−3^. (f) QE profiles. (g) Nyquist curves. (h) Recombination rate distribution.

To further improve the device performance of MAPbI_3_ PSC, we introduced a 10-nm interlayer of Nb_2_O_5_ between perovskite and ETL. The corresponding *J*–*V* curve reveals that interfacial modification boosts device performance, particularly *V*_OC_ and FF (Fig. 4a). The Nb_2_O_5_-based cell yields a PCE of 22.67% compared to 19.35% for the unmodified cell. The modified device shows a closely similar *J*_SC_ value and comparable QE spectrum (Fig. 4f), implying that the improvement arises from enhanced band alignment and carrier dynamics rather than additional light harvesting. The conduction band edge of Nb_2_O_5_ is between the MAPbI_3_ and SnO_2_ layer, which can reduce the CBO between them and promote electron extraction (Fig. 4b and c). Moreover, the electric field curve further shows a strong peak at the SnO_2_/Nb_2_O_5_ interface, assisting carrier transfer (Fig. 4d). Fig. 4e exhibits the Mott–Schottky plot of the Nb_2_O_5_-engineered device extracted from capacitance–voltage simulation under dark conditions. The voltage-axis intercept of the linear region was used to estimate built-in potential (*V*_bi_). This plot gives a built-in potential value of 1.4 V due to the changing electrostatic and capacitance behaviour after interlayer incorporation.

**Fig. 4:**
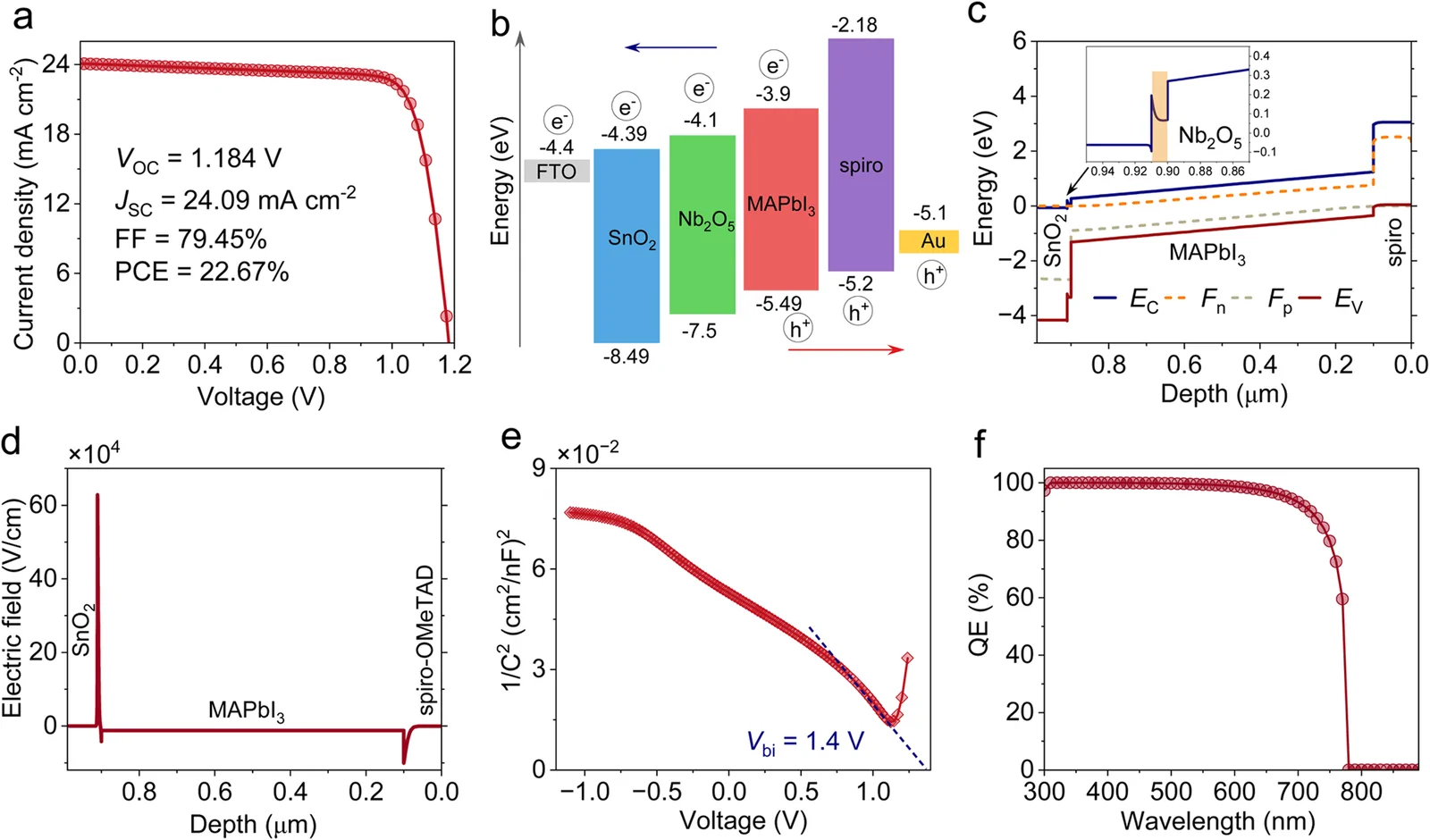
Device enhancement using interfacial engineering at the SnO_2_/MAPbI_3_ junction. (a) *J*–*V* plot of PSC with a 10-nm Nb_2_O_5_ interlayer. (b) Energy level frontiers after adding the interlayer. (c) Band structure diagram. Inset: zoomed in diagram at the SnO_2_/Nb_2_O_5_ interface. (d) Electric field distribution. (e) Mott–Schottky plot. (f) QE curve.

Fig. 5a–d exhibit the contour plots of photovoltaic parameters at different defect densities at the SnO_2_/Nb_2_O_5_ and Nb_2_O_5_/MAPbI_3_ interfaces. As observed, the Nb_2_O_5_/MAPbI_3_ interfacial defect density largely controls the device performance. Because the junction between n-type and p-type materials is more vulnerable to hole–electron recombination. Whereas, the identical type junction has a restricted hole population. The increased defect density from 10^12^ cm^−2^ to 10^15^ m^−2^ strongly suppresses *V*_OC_ from 1.254 V to 1.096 V and PCE from 23.83% to 20.95%. While *J*_SC_ remains nearly constant 24.08 mA cm^−2^, as supported by the *J*–*V* plots in Fig. 5e. The QE results also corroborated with this trend, where photocarriers remain efficiently generated and collected under the investigated range (Fig. 5f). Fig. 5g reveals a slight reduction in the Nyquist curve after increasing interfacial defect density owing to unchanged recombination in bulk perovskite layer (Fig. 5h).

**Fig. 5:**
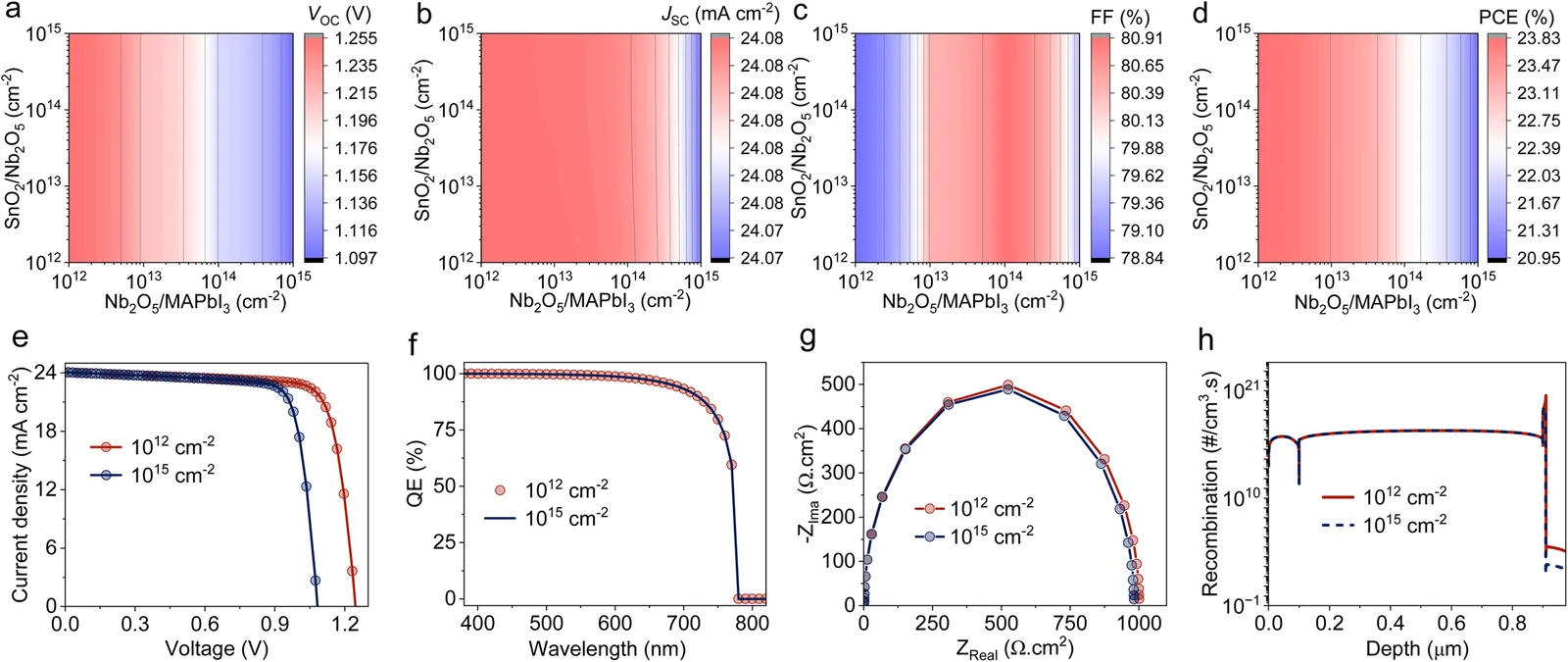
Optimizing the defect density at the SnO_2_/Nb_2_O_5_ and Nb_2_O_5_/MAPbI_3_ interfaces. The contour maps for (a) *V*_OC,_ (b) *J*_SC_, (c) FF, and (d) PCE of devices. (e) The *J*–*V* plots of devices at low and high interfacial defect densities at a constant MAPbI_3_/spiro-OMeTAD defect density of 10^12^ cm^−2^. (f) QE spectra. (g) Nyquist curves. (h) Recombination rate profiles.

Fig. 6a–d show the contour plots of photovoltaic parameters of Nb_2_O_5_-based PSCs changing with *R*_S_ and *R*_SH_. These resistances mostly affect *J*_SC_ and FF, whereas *V*_OC_ remains unchanged, particularly with altering *R*_S_. Higher *R*_S_ induces voltage losses during current flow, thus damaging the *J*–*V* characteristics and minimizing the output power. Meanwhile, higher *R*_SH_ is preferable to reduce current leakage pathways. The optimized Nb_2_O_5_ device achieves 25.196% efficiency with 1.248 V *V*_OC_, 24.128 mA cm^−2^*J*_SC_, and 83.665% FF at 1.0 Ω cm^2^ and 5000 Ω cm^2^ of *R*_S_ and *R*_SH_, respectively. The distinction between two cases for parasitic resistances is shown in Fig. 6e. The coupled low *R*_S_ and high *R*_SH_ generate an enhanced *J*–*V* plot due to a significant increase in the FF. While, QE profiles show a comparable response throughout entire visible range (Fig. 6f). Fig. 6g displays the capacitance–frequency (*C*–*f*) curves of the Nb_2_O_5_ devices under AC perturbation. The low *R*_S_/high *R*_SH_ configuration reveals a stable capacitive pattern due to a rapid electrical response and limited resistive distortion. Nyquist plots demonstrate significant enlargement for low *R*_S_/high *R*_SH_ configuration (Fig. 6h), indicating a reduction in recombination losses. This Nyquist plot improvement may also originate from changes in the *R*_SH_.

**Fig. 6:**
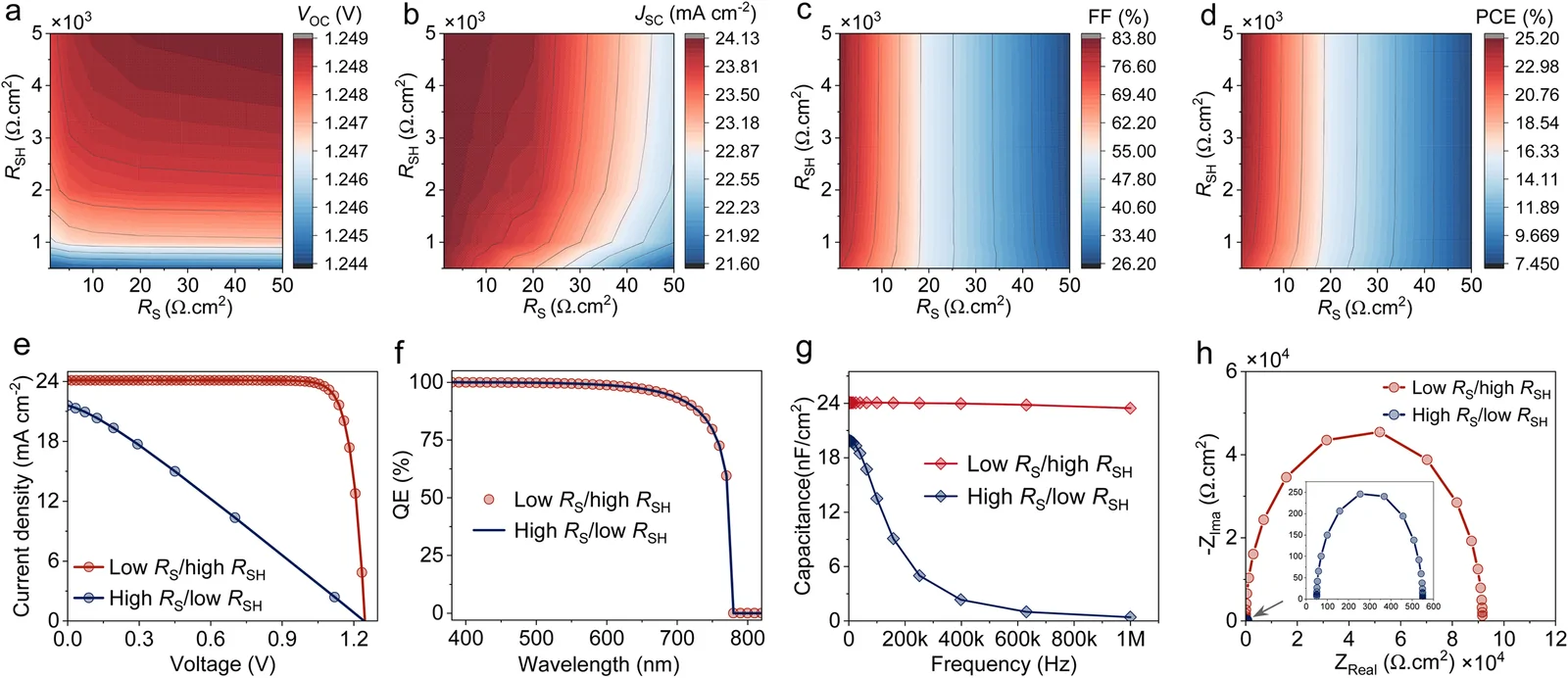
Analysis of the internal resistive losses in the optimized PSCs. The contour plots for (a) *V*_OC,_ (b) *J*_SC_, (c) FF, and (d) PCE of devices. (e) The *J*–*V* plots of devices at low and high parasitic resistances. (f) QE plots. (g) *C*–*f* characteristics under AC bias. (h) Nyquist curves.

Fig. 7a exhibits the impact of operating temperature on *J*–*V* curves for the optimized Nb_2_O_5_-based PSCs. As shown in Fig. 7b and c, increasing the temperature to 400 K mainly suppresses the V_OC_ and FF to 1.157 V and 78.977%, respectively. This reduction can be related to increasing the dark saturation current, which limits the voltage at light conditions. In addition, higher temperatures slightly reduce the Nyquist plot (Fig. 7d), indicating transport and recombination effects. However, the optimized device shows low reduction in electrical properties with increasing temperature to 400 K, as shown in Fig. 7e.

**Fig. 7:**
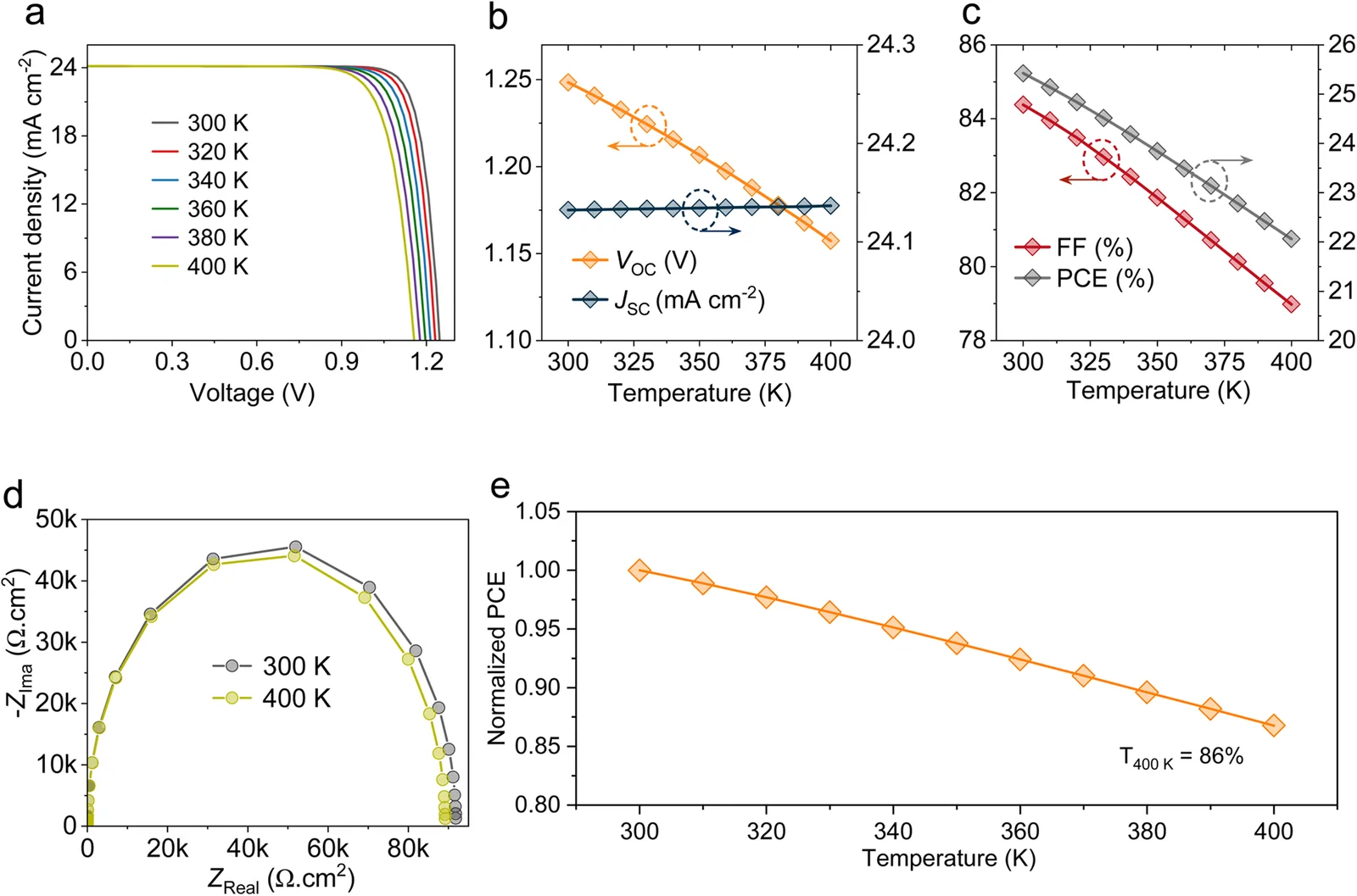
Temperature-dependent device performance of the simulated PSCs. (a) *J*–*V* curves with varying temperatures from 300 K to 400 K with 20 K steps. (b) Variations of *V*_OC_ and *J*_SC_. (c) Dependency of FF and PCE on temperature. (d) Nyquist plots. (e) Tracking PCE change with working temperature.

Fig. 8a shows the *J*–*V* plots of optimized PSCs with and without the Nb_2_O_5_ interlayer. The results reveal interfacial engineering mainly improves the *V*_OC_, while maintaining *J*_SC_ constant. The unchanged QE results also confirm this pattern (Fig. 8b); thus, Nb_2_O_5_ addition has no effect on optical absorption and carrier collection. The modified device induces higher *V*_OC_ thanks to better band alignment and modified carrier distribution, thereby limiting recombination processes at interfaces and maintaining high QFLS and *V*_OC_. The modification achieved a maximum efficiency of 25.196% in single-junction PSC in comparison to the 22.663% for the device Nb_2_O_5_ interlayer. Fig. 8c reveals a minor increase in the impedance plot after Nb_2_O_5_ addition, which may arise from the reduction of interfacial recombination losses at the SnO_2_/Nb_2_O_5_ interface (Fig. 8d). Fig. 8e and f depict the electric field localization within PSCs before and after the Nb_2_O_5_ insertion. The simulated device with Nb_2_O_5_ interlayer shows a stronger localized positive field than that of the unmodified device. The maximum electric-field value for the Nb_2_O_5_-based device improves from 6 × 10^5^ V cm^−1^ (Fig. 4d) in the baseline configuration to 1.2 × 10^6^ V cm^−1^ after optimization (Fig. 8e). This increment is due to changes in local space charge and potential gradients, which may increase the total built-in potential. Table 3 displays the optimization steps of the proposed PSC with interfacial engineering. Initially, the calibrated PSC with the experimentally fabricated device attained an efficiency of 14.04%, then improved to 19.31% following MAPbI_3_ perovskite optimization. After that, the device performance is further enhanced to 22.66% by introducing a 10-nm Nb_2_O_5_ interlayer. This PCE increase is due to improving the *V*_OC_ from 1.046 V to 1.184 V after Nb_2_O_5_ insertion. Finally, further optimization of interface defect density, *R*_S_, and *R*_SH_ increased the PCE to 25.19%, implying the benefits of interfacial engineering and electrical parameter optimization in the simulated PSC. SCAPS-1D simulations predict the enhanced PV parameters due to Nb2O5 insertion; however, experimental validation for the proposed PSC are necessary to confirm the simulated performance and the effectiveness of the Nb_2_O_5_ layer in modifying the SnO_2_/perovskite interface.

**Fig. 8:**
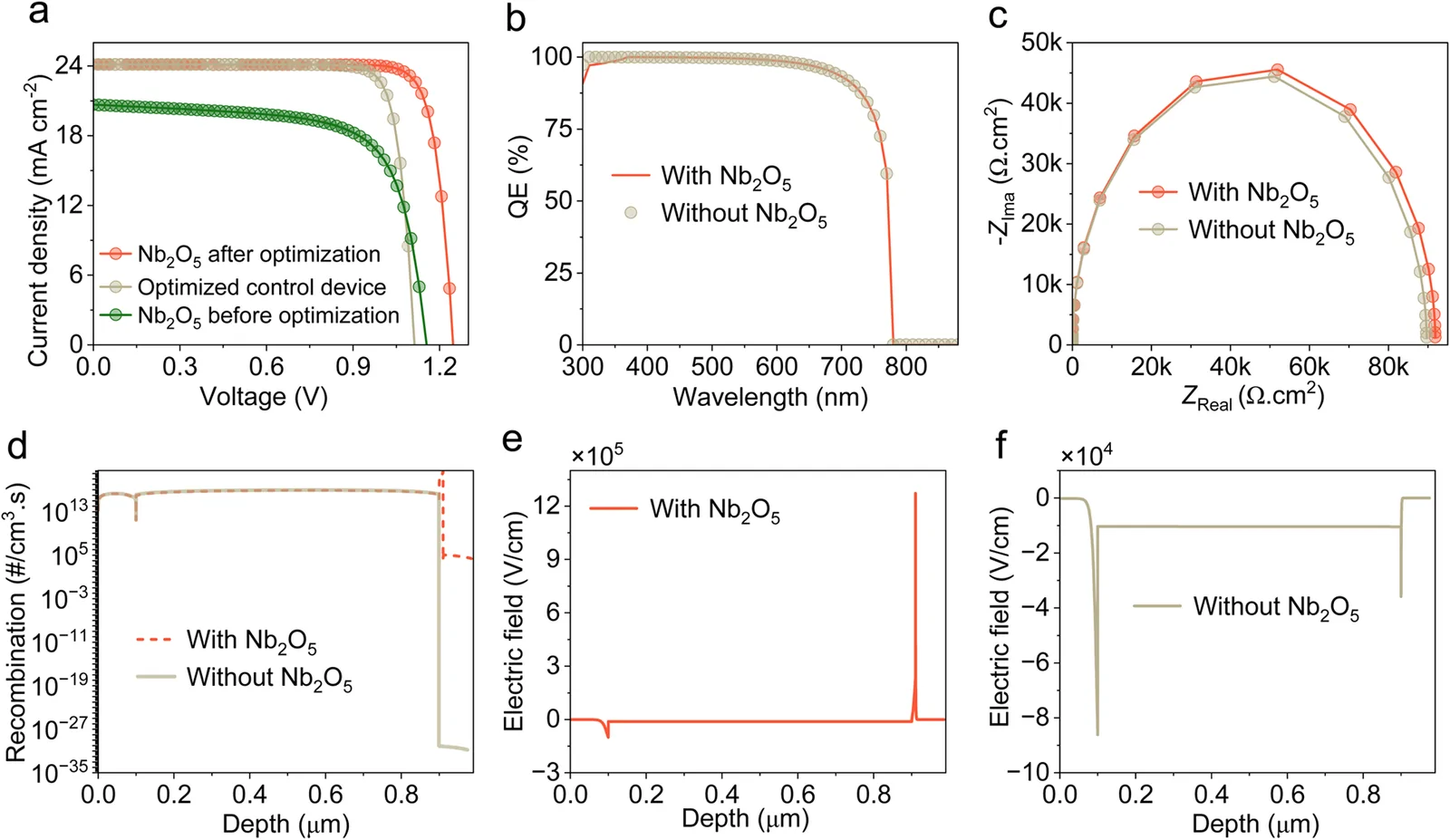
Comparison between optimized device performance before and after Nb_2_O_5_ insertion. (a) The *J*–*V* curves. Control sample is the optimized device without Nb_2_O_5_. (b) QE spectra. (c) Nyquist curves. (d) Recombination rate curves. (e) Electric field distribution of Nb_2_O_5_-engineered device. (f) Electric field distribution of the unmodified device.

**Table 3:** The simulated device parameters during simulations

Parameters	Investigated range	Optimized value	*V*_OC_ (V)	*J*_SC_ (mA cm^−2^)	FF (%)	PCE (%)
Initial calibrated device	—	—	1.034	20.61	65.82	14.04
Bulk defect density [cm^−3^]	10^14^–10^18^	10^14^	1.042	20.79	75.07	16.28
Layer thickness [µm]	0.2–0.8	0.8	1.046	23.79	72.76	18.12
Hole mobility [cm^2^ V^−1^ s^−1^]	10^−3^ – 20	20	1.047	23.76	72.62	18.08
Acceptor density [cm^−3^]	10^13^–10^17^	10^13^	1.046	24.07	76.66	19.31
Adding 10-nm Nb_2_O_5_ interlayer	—	—	1.184	24.09	79.45	22.66
Interface defect density [cm^−2^]	10^12^–10^15^	10^12^	1.254	24.08	78.84	23.82
Series resistance [Ω cm^2^]	1.0–50	1.0	1.254	24.11	80.40	24.33
Shunt resistance [Ω cm^2^]	500–5000	5000	1.248	24.12	83.66	25.19

The obtained *PV* parameters of the optimized Nb_2_O_5_-based PSC were compared with recently reported PSC structures (Table 4). The device yielded a predicted PCE of 25.19%, which exceeds several efficiencies reported for the Nb_2_O_5_- and Al_2_O_5_-based PSCs. These differences in device performance are due to variation of perovskite composition, layer configuration (n-i-p or p-i-n), and material properties.

**Table 4:** A comparison between this simulated work and recently reported PSCs

Structure	Year	*V*_OC_ (V)	*J*_SC_ (mA cm^−2^)	FF (%)	PCE (%)	Ref.
ITO/Nb_2_O_5_/MA_2/6_FA_4/6_Pb(Br_1/6_I_5/6_)_3_/spiro-OMeTAD/Au	2017	1.06	24.69	71.10	18.59	34
ITO/SnO_2_/Al_2_O_3_/MAPbI_3_/spiro-OMeTAD/Au	2019	1.14	22.87	73.14	19.10	31
FTO/Nb_2_O_5_/CsSn_0.5_G_e0.5_I_3_/Spiro-OMeTAD/Au	2024	1.27	26.34	83.85	28.25	35
ITO/Me-4PACz/SiOx/Cs_0.05_(FA_0.90_MA_0.10_)_0.95_(I_0.8_Br_0.2_)_3_/C_60_/AlO_*x*_/SnO_*x*_/Ag	2024	1.19	20.50	83.00	20.10	36
FTO/SnO_2_/AlCl_3_/perovskite/PEAI/spiro-OMeTAD/Au	2025	1.19	26.86	82.70	26.54	9
FTO/Nb_2_O_5_/perovskite/HTL/Au	2025	1.15	25.59	78.00	23.11	37
ITO/SnO_2_/NbO_*x*_/perovskite/spiro-OMeTAD/Au	2025	1.17	24.87	81.31	23.64	38
ITO/Ph-4PACz/perovskite/3MTPAI + PDAI_2_/C_60_/BCP/CrO_*x*_/Nb_2_O_5_/Ag	2026	1.19	25.37	85.35	25.85	39
**FTO/SnO_2_/Nb_2_O_5_/MAPbI_3_/spiro-OMeTAD/Au**	**2026**	**1.248**	**24.12**	**83.66**	**25.19**	**This work**

## 4. Conclusion

In summary, we propose an efficient interfacial engineering processed the SnO_2_/MAPbI_3_ interface by an ultrathin Nb_2_O_5_ layer for PSCs application. The planar PSC based on the Nb_2_O_5_ interlayer achieves an efficiency as high as 25.19% compared to the unmodified device with only 22.66% efficiency. The high photovoltaic performance is attributed to the improved interface quality and favorable energy-level alignment of Nb_2_O_5_ for tuning electron-injection and/or charge-recombination dynamics. The optimized Nb_2_O_5_-based cell attained a *V*_OC_ of 1.248 V compared with 1.114 V for the optimized reference device without Nb_2_O_5_. Upon introducing a 10-nm-thick Nb_2_O_5_ interlayer, the *V*_OC_ parameter increases by 134 mV due to a reduction of nonradiative recombination and conduction band offset at the SnO_2_/MAPbI_3_ junction. The simulated *V*_OC_ of 1.248 V at a bandgap of 1.59 eV corresponds to a voltage deficit loss of 0.342 V. The predicted voltage approximates the estimated value of the radiative limit; however, this result is sensitive to the assumed material parameters, defect densities, and recombination mechanisms. The combined parameter analyses further show that the beneficial effect of increasing perovskite thickness depends on low defect density, while good collection efficiency requires sufficient hole mobility. Moreover, the optimization of Nb_2_O_5_ interfacial defects significantly reduces carrier recombination within the PSCs, while also acting as an electron-selective interlayer.

## Conflicts of interest

The authors declare no conflicts of interest.

## Data Availability

The data will be available from the corresponding author on reasonable request.
